# Disaster Risk Science: A Geographical Perspective and a Research Framework

**DOI:** 10.1007/s13753-020-00296-5

**Published:** 2020-08-21

**Authors:** Peijun Shi, Tao Ye, Ying Wang, Tao Zhou, Wei Xu, Juan Du, Jing’ai Wang, Ning Li, Chongfu Huang, Lianyou Liu, Bo Chen, Yun Su, Weihua Fang, Ming Wang, Xiaobin Hu, Jidong Wu, Chunyang He, Qiang Zhang, Qian Ye, Carlo Jaeger, Norio Okada

**Affiliations:** 1 State Key Laboratory of Earth Surface Processes and Resource Ecology, Beijing Normal University, Beijing, 100875 China; 2 Key Laboratory of Environmental Change and Natural Disasters, Ministry of Education, Beijing Normal University, Beijing, 100875 China; 3 Academy of Disaster Reduction and Emergency Management, Ministry of Emergency Management and Ministry of Education, Beijing, 100875 China; 4 Faculty of Geographical Science, Beijing Normal University, Beijing, 100875 China; 5 Academy of Plateau Science and Sustainability, People’s Government of Qinghai Province and Beijing Normal University, Xining, 810016 China; 6 Global Climate Forum, 10178 Berlin, Germany; 7 Disaster Prevention Research Institute, Kyoto University, Kyoto, 611-0011 Japan

**Keywords:** Disaster system, Disaster science, Disaster technology, Disaster governance, Research framework, Research frontiers

## Abstract

In this article, we recall the United Nations’ 30-year journey in disaster risk reduction strategy and framework, review the latest progress and key scientific and technological questions related to the United Nations disaster risk reduction initiatives, and summarize the framework and contents of disaster risk science research. The object of disaster risk science research is the “disaster system” consisting of hazard, the geographical environment, and exposed units, with features of regionality, interconnectedness, coupling, and complexity. Environmental stability, hazard threat, and socioeconomic vulnerability together determine the way that disasters are formed, establish the spatial extent of disaster impact, and generate the scale of losses. In the formation of a disaster, a conducive environment is the prerequisite, a hazard is the necessary condition, and socioeconomic exposure is the sufficient condition. The geographical environment affects local hazard intensity and therefore can change the pattern of loss distribution. Regional multi-hazard, disaster chain, and disaster compound could induce complex impacts, amplifying or attenuating hazard intensity and changing the scope of affected areas. In the light of research progress, particularly in the context of China, we propose a three-layer disaster risk science disciplinary structure, which contains three pillars (disaster science, disaster technology, and disaster governance), nine core areas, and 27 research fields. Based on these elements, we discuss the frontiers in disaster risk science research.

## Introduction

2019 was the 30th anniversary of the United Nations (UN) global disaster risk reduction (DRR) initiatives. On 11 December 1987, the UN General Assembly announced plans to organize the International Decade for Natural Disaster Reduction (IDNDR) in the 1990s. On 23–27 May 1994, the First World Conference on Disaster Reduction was held in Yokohama, Japan. Its output, the *Yokohama Strategy and Plan of Action for a Safer World* (IDNDR 1994), suggested that disaster prevention, preparedness, mitigation, and relief are the key measures needed to build a safer world and realize sustainable development. In November 2000, the 54th UN General Assembly decided to implement the International Strategy for Disaster Reduction (ISDR), and set up a Secretariat for coordination. On 18–22 January 2005, the Second World Conference on Disaster Reduction was held in Kobe, Japan. The *Hyogo Framework for Action 2005*–*2015* (UNISDR 2005) was adopted, which emphasized building the resilience of nations and communities to disasters. On 14–18 March 2015, the Third UN World Conference on Disaster Risk Reduction was held in Sendai, Japan. Its official output, the *Sendai Framework for Disaster Risk Reduction 2015*–*2030* (UNISDR 2015), exclusively outlined four priorities: understand disaster risk, strengthen disaster risk governance to manage disaster risk, invest in disaster risk reduction for resilience, and enhance disaster preparedness for effective response and to Build Back Better in recovery, rehabilitation, and reconstruction. The Sendai Framework also called for enhanced international collaboration to build a global DRR partnership to face the challenges of climate change and achieve the sustainable development goals. So far, it has been a 30-year endeavor of all countries, sectors, and stakeholders to seek for the scientific strategies, effective methods, advanced technologies, and powerful measures that promote DRR.

The fundamental motivation of disaster risk science and technology (S&T) development is to protect life and assets and achieve sustainable socioeconomic development. The scientific community, primarily Earth sciences researchers, has responded actively to the UN global DRR initiatives, and has systematically provided S&T support. During the 30 years of UN global initiatives, from hazard mitigation to disaster reduction and then to DRR, the academic community has conducted a series of systematic and integrated research efforts to address global, regional, subregional, country, local, and community needs. The topics have covered the entire cycle of disaster management (prevention, preparedness, emergency response, recovery, and reconstruction) and regional actions for DRR from the perspective of Earth system science. The scope and focus of disaster risk science are summarized in subsequent subsections: Sects. 1.1 and 1.2.

### Hazards, Disasters, Disaster Risks, and Their Linkages

The academic community has not reached a consensus on the concepts of hazard, disaster, and disaster risk. Under the Sendai Framework for Disaster Risk Reduction 2015–2030, the United Nations proposed a terminology system that has been widely adopted by researchers and practitioners: Hazard is “a process, phenomenon or human activity that may cause loss of life, injury or other health impacts, property damage, social and economic disruption or environmental degradation” (UNDRR 2017). Hazards include biological, environmental, geological, hydrometeorological and technological processes and phenomena (UNISDR 2015). Disaster is “a serious disruption of the functioning of a community or a society at any scale due to hazardous events interacting with conditions of exposure, vulnerability and capacity, leading to one or more of the following: human, material, economic and environmental losses and impacts” (UNDRR 2017). Disaster risk is “the potential loss of life, injury, or destroyed or damaged assets that could occur to a system, society or a community in a specific period of time, determined probabilistically as a function of hazard, exposure, vulnerability and capacity” (UNDRR 2017).

**Fig. 1:**
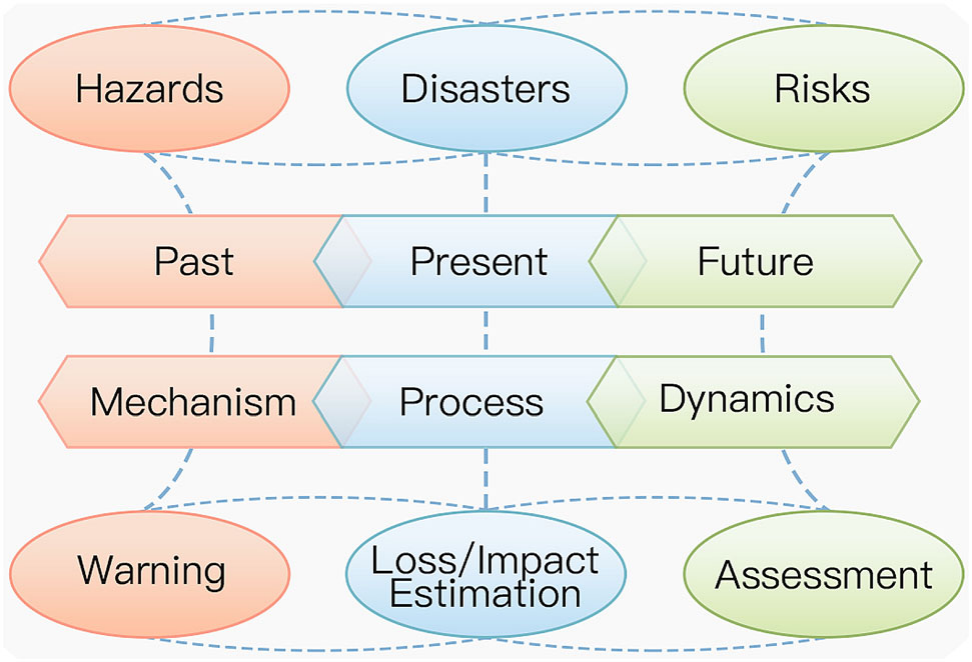
Relationships between hazards, disasters, and risks

Figure 1 presents a framework that elaborates the linkages and differences among hazards, disasters, and disaster risks (Shi 2018). For hazards, the observation, monitoring, and measurement of hazards help to reveal the spatial–temporal patterns and formation mechanisms, and develop statistical and/or process-based models for prediction, forecasting, and early warning. On these bases, the formation processes of individual disaster events can be modeled, and their consequences can be estimated, including human casualties, losses in property and damage to resources and environment, and further economic, political, cultural, societal, and ecosystem effects. The estimation of disaster impacts in a region as a whole through time (regional disasters) can be obtained from statistical analysis of regional disaster pattern and construction of indices and models. For risks, by understanding the hazard mechanism and disaster process of a regional disaster system, future risk of disasters at the event and regional scales under difference scenarios can be assessed with disaster risk models and modeling that integrates hazard forecasting modules and disaster impact estimation modules.

### The Disaster System

**Fig. 2:**
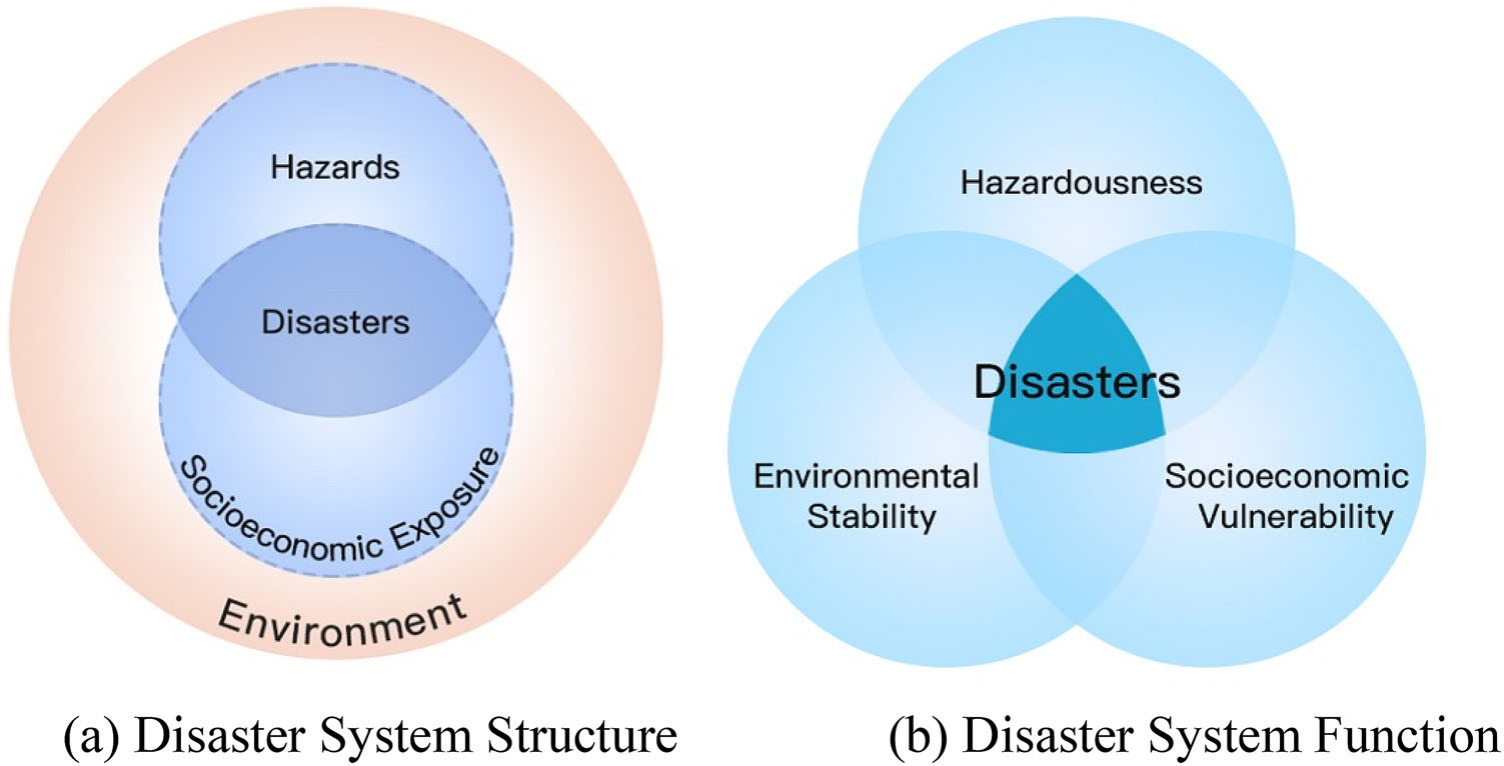
A conceptual model of disaster system. *Source* Adapted from Shi (1991, 2005)

In order to deepen understanding of the relationship between hazards and the formation of disasters, the concept of disaster system (DS) has been proposed (Shi 1991). The DS is regarded as the object of disaster risk science research, and has both structure and function (Fig. 2). The structure presents the elements of a DS. To Ma and Gao the DS consists of planet Earth, human beings, and atmospheric, geological, hydrometeorological, and biological hazards (Ma and Gao 1990); or a DS can be described as an Earth surface metamorphic system consisting of the geographical environment, hazards, socioeconomic exposure, and disaster (losses) (Shi 1991) (Fig. 2a); or alternatively as a coupled system of Earth system, human system, and construction system (Mileti 1999).

The interlinkages and interactions among the elements determine the function of the DS (Fig. 2b) (Shi, Wang, et al. 2014). Disaster vulnerability in a broader sense contains resilience and adaptability (Turner et al. 2003). Key function attributes of the core elements of a disaster system (Df) therefore include environmental stability (or sensitivity) (S), hazardousness (H), and vulnerability of the exposed units (V). The interactions of those attributes determine the state and function of a disaster system, Df = S ∩ H ∩ V (Shi 2005). Burton et al. stated that vulnerability plays a key role in the formation of disasters (Burton et al. 1993). Wisner emphasized the interaction of hazards and exposure during the formation of disasters (Wisner et al. 2004): No hazard, no disaster; no exposure, no disaster, either. This is exactly the reason that the second edition of *At Risk: Natural Hazards, People’s Vulnerability and Disasters* has revised its conceptual framework from *R*(*isk*)=* H *+* V* to *R *=* H *×* V*, which clarifies the necessary condition of both hazard and exposure (socioeconomic vulnerability) in disasters.

The regional geographical environment within which hazards form determines not only the spatial distribution of exposure, but also influences the complexity of hazards and further alters hazard intensity. Such influences could be critical in triggering completely different multi-hazards, disaster chains, and disaster compounding events. This can be demonstrated by the dramatic difference between the 2008 Wenchuan Earthquake in China that occurred in a mountainous region, and the 2011 Eastern Japan Great Earthquake and Tsunami that occurred off-shore. The Wenchuan Earthquake triggered a large number of rock-falls and landslides, which together with rainstorms further induced debris flows and quake lakes, which formed a typical disaster chain of earthquake–rock fall and landslide–rainstorm–debris flow–quake lake (Shi 2008a). The East Japan Earthquake, by contrast, triggered a tsunami, which in turn damaged the nuclear power plant in Fukushima, forming a typical Natech (Natural disaster-triggered technological accident) disaster chain of earthquake–tsunami–nuclear power plant failure–radiative accidents (Okada et al. 2011). Their difference vividly illustrates the critical roles of the geographical environment in modifying local hazards. It also reveals that the structure of a disaster system determines its function and regional features.

## Advances in Disaster Risk Science Research

In this section, we review the major scientific advances in disaster risk research, in terms of our understanding of each of the key elements—hazards, disaster, disaster risks and disaster response, as well as integrated studies.

### Hazard Study in Disaster Risk Science

Studies of hazards have always been fundamental in disaster risk science research. Their focus is to deepen our understanding of the causes and formation mechanism of hazards, so as to improve forecasting capability and accuracy and, in turn, the effectiveness of early warning. Researchers from the fields of seismology, meteorology, geology, hydrology, biology, and geography have conducted long-term systematic research on the formation mechanism and processes, dynamics, and causes of various types of natural hazards. In addition to the systematic research within single disciplinary fields, transdisciplinary, comprehensive studies have also been conducted about earthquakes, tropic cyclones, landslides, debris flows, floods, diseases and pests, wildfires, droughts, land degradation, and desertification. The launch of the IDNDR greatly stimulated hazard studies. Several important academic journals were founded, including *Natural Hazards* (1988 in the United States), *Environmental Hazards* (1999 in the United Kingdom), *Natural Hazards Review* (2000 in the United States), and *Natural Hazards and Earth System Sciences* (2001 in Germany). There are also many hazard study books such as *Recent Studies in Geophysical Hazards* (El-Sabh et al. 1994), *Natural Hazards* (Chen and Shi 2013), and the *Atlas of Natural Disasters in China* published bilingually in Chinese and English (Zhang 1992).

Recent advances within the focus of hazard studies include, for example, further understanding of earthquake occurrence, which has occurred by connecting slow earthquakes to huge earthquakes (Obara and Kato 2016), earthquake forecast (Huang 2015), and early-warning system improvement (Cochran and Husker 2019). Sea-surface temperature change and its impact on tropic cyclone frequency and intensity is explored by Emanuel (2017). Other examples include the impact of global warming (Yamaguchi et al. 2020) and rising sea-level on tropical cyclones and storm surges (Calafat and Marcos 2020), and their influence on costal residents’ migration (Hauer 2017). The linkages between climate change and hydrometeorological hazards (for example, floods and heatwaves) and their secondary hazards (for example, landslides and debris flows), mostly concentrate on the altered frequency and intensity of hazards driven by climate change (Stott 2016; Blöschl et al. 2019), and have spurred the construction and improvement of early-warning systems (Schiermeier 2018). The connection between climate change and environmental hazards such as droughts, wildfires, land degradation, and desertification are examined by Mazdiyasni and AghaKouchak (2015), especially the cryosphere change in higher elevation regions and its corresponding impact on the water supply of oases in arid basins (Pritchard 2019). Besides these single hazard studies, multi-hazards, hazard chains or cascading hazards (Walter et al. 2019), and hazard compounds (Bevacqua et al. 2019) are attracting increasing attention.

### Disaster Study in Disaster Risk Science

Studies of hazards have been accompanied by research on disasters, and the focus of the latter has been on the loss-formation mechanism, and evaluation of disaster losses and impacts. Special attention has been paid to the critical roles of human and socioeconomic factors in the formation of disasters, and their amplification on the effects of natural and environmental hazards. Journals, including *Disasters* (founded in 1977 in the United Kingdom), the *Journal of Catastrophology* (founded in 1986 in China), and *Progress in Disaster Science* (founded in 2018 in the Netherlands), have promoted the advance in this field.

Key topics in this field, repeatedly published in the journals mentioned above, include, for instance, the global impact of volcanic eruption (Papale and Marzocchi 2019); the spatial–temporal distribution of urban heat wave exposure and loss (Yang et al. 2019); climate change and flood vulnerability (Jongman et al. 2015); socioeconomic impact assessment of climate change (Carleton and Hsiang 2016; Su et al. 2018); the damage of climate extremes to road infrastructure and transportation (Wang et al. 2019), among many others. These studies have largely improved the methodology and models available for disaster loss assessment, particularly for climate trend effects and climate change-related disasters, and have significantly advanced disaster risk science.

Many important books have also been published in this field, including *The Environment as Hazard* (Burton et al. 1993), *Major Natural Disasters in China and the Mitigation Countermeasures (General Issues)* (Ma 1994), *Disasters by Design* (Mileti 1999), the *Atlas of Natural Disaster Systems of China*, which appears in a bilingual Chinese and English edition (Shi 2003), *At Risk: Natural Hazards, People’s Vulnerability and Disasters* (Wisner et al. 2004), and *Natural Disasters in China* (Shi 2016), among others. These publications have mostly centered on the relationship between resource exploitation and natural hazards and disasters from the perspective of human activity, and have paid close attention to the role of human and socioeconomic factors in the formation of disasters, all contributing in important ways to the development of disaster risk science research.

### Risk Study in Disaster Risk Science

Studies on risks have involved the extension of disaster studies to meet the urgent needs of risk reduction, with foci on disaster risk assessment, simulation, and governance approaches. *Risk Analysis* (founded in 1981 in the United States) has concentrated on the mathematical models and modeling of disaster risk. Other journals such as *Jàmbá: Journal of Disaster Risk Studies* (founded in 2006 in South Africa), and *Geomatics, Natural Hazards & Risk* (founded in 2010 in the United Kingdom) have also published systematic studies on disaster risk. In 2010, the *International Journal of Disaster Risk Science* was founded in China, which for the first time formally put forward the term “disaster risk science.”

Advances in risk studies in recent years include, for example, seismic risk simulation using scenario ensembles (Robinson et al. 2018) and risk reduction (Tucker 2013), global trends of tropical cyclone risks (Peduzzi et al. 2012), climatic and socioeconomic controls in coastal flood risks (Vousdoukas et al. 2018), climate change and global flood risk and its reduction (Kundzewicz et al. 2018; Ward et al. 2015; Aerts et al. 2018), avalanche risk (Ballesteros-Cánovas et al. 2018), wild fire risk (Radeloff et al. 2018), and multi-hazard risk (Koks et al. 2019). There has been an increasing concern about networked risk (Helbing 2013) and compound event risks (Zscheischler et al. 2018), and crop synchronized failure risk (Gaupp et al. 2020), and so on.

There are also many books published in the risk field, for example, *Integrated Catastrophe Risk Modeling* (Amendola et al. 2013), *Hazards, Risks, and Disasters in Society* (Collins et al. 2014), *The Social Roots of Risk: Producing Disasters, Promoting Resilience* (Tierney 2014), the *World Atlas of Natural Disaster Risk* (Shi and Kasperson 2015), *Risk Modeling for Hazards and Disasters* (Michel 2018), among many others. In the context of China, representative works include *Mountainous Disaster Forming Mechanism and Risk Control in Wenchuan Earthquake* (Cui et al. 2011), the *Atlas of Natural Disaster Risk of China* (Shi 2011a), *Integrated Risk Governance: Science, Technology and Demonstration* (Shi 2011b), *Integrated Risk Governance* (Shi, Jaeger, et al. 2012), the *Atlas of Environmental Risks Facing China Under Climate Change* (Tang and Ge 2018), *Disaster Risk Science* (Shi 2018), and so on. These publications have substantially pushed forward the development of disaster risk science research in China and globally, deepened understanding of disaster risk, particularly for climate change, multi-hazards, disaster chains, and disaster compounds, and provided scientific support to risk reduction plans.

### Disaster Response Study in Disaster Risk Science

Disaster response includes two pillars: the response system for individual disaster events, namely, prevention, preparedness, emergency response, recovery and reconstruction; and the response system for regional disasters, namely the combination of prevention, consilience, relief, and integrated disaster risk governance.

In response to the United Nations call for global hazard mitigation, disaster reduction, and DRR, several scientific programs have been launched, a set of new academic journals have been founded, and new research output in the form of journal articles and books have been published. In 2008, the International Council for Science (ICSU) launched the *Integrated Research on Disaster Risk* (IRDR) program (ICSU 2008). The International Human Dimension Program (IHDP) launched the Integrated Risk Governance (IRG) core science project (Shi, Jaeger, et al. 2012). In 2015, IRG formally enrolled in the Future Earth program. In this field, representative academic journals include *Geological Hazard and Control* (founded in 1990 in China, and renamed *The Chinese Journal of Geological Hazard and Control* in 1991), *Disaster Prevention and Management* (founded in 1992 in the United States), the *Journal of Flood Risk Management* (founded in 2008 in the United Kingdom), the *International Journal of Disaster Resilience in the Built Environment* (founded in 2010 in the United Kingdom), and *International Journal of Disaster Risk Reduction* (founded in 2012 in the Netherlands). These journals have provided important platforms for academic discussion of disaster response.

The latest progress in disaster response study, for instance, has focused on: the function of preparedness in disaster response (McNutt 2015); optimization of emergency coping resources (Hanson and Roberts 2005); emergency relocation and social capital (Hikichi et al. 2017); disaster insurance’s plausible roles (Surminski et al. 2016), and so on. Other studies call for effective adaptation to climate change to reduce disaster risk (Jongman 2018), particularly increased adaptation in the private sector (Goldstein et al. 2019), prevention capacity improvement in the wildland-urban interface (Calkin et al. 2014) and coastal regions (Barbier 2014), protection of the environment and ecosystem services (Reyers et al. 2015), adjustments in human exposure in quantity and distribution (Spears 2015).

Representative publications in this field include *Large*-*scale Disasters: Prediction, Control, and Mitigation* (Gad-el-Hak 2008), *Managing the Risks of Extreme Events and Disasters to Advance Climate Change Adaptation* (IPCC 2012), *Hazards, Risks, and Disasters in Society* (Collins et al. 2014), *Integrated Disaster Science and Management: Global Case Studies in Mitigation and Recovery* (Samui et al. 2018), and *Science and Technology in Disaster Risk Reduction in Asia: Potentials and Challenges* (Shaw et al. 2018).

Important concentrations of research in the disaster response field include the improvement of disaster and risk management, implementation of disaster reduction strategies, improvement of early-warning technology and information services for effective response, an improved scientific basis for disaster insurance, and disaster aid for developing countries. These research nodes have greatly promoted scientific research in disaster response, provided solid S&T support to effective disaster response, and supplied evidence for the UN’s strategy for reducing disaster impact and DRR.

## A Framework for Disaster Risk Science Research

Developing disaster risk science is imperative to meet the need for DRR, and is the product of and basis for implementing the *Sendai Framework for Disaster Risk Reduction 2015*–*2030* of the United Nations (Aitsi-Selmi et al. 2016). The terminology system is not complete, and some of the terms overlap or are contradictory (Kelman 2018). This situation requires scholars to learn from other disciplines and improve the precision of their terminology in order to establish an interdisciplinary group of disaster risk science researchers.

Disaster risk science is the discipline that studies the hazard mechanism, disaster process, dynamics modeling, spatial–temporal patterns of disaster impact (effects and losses), emergency response, and risk governance paradigms of disaster systems. It is a multi-, cross-, and transdisciplinary field. The structure of disaster risk science is different from that of the Earth science system, which is divided into Earth spheres (such as geology, geography, atmospheric science, oceanology and ecology, and so on), or divided by basic science linkage (such as geomathematics, geophysics, geochemistry, geometrics, digital Earth, and so on). By contrast, the division of disaster risk science is closer to that of the study of the cryosphere (Qin et al. 2017). Disaster risk science includes not only basic (including applied basic) studies, namely, theory and methodology, but also systematic application-oriented research and development (R&D), namely, response technologies and governance approaches and their integration. Because the disaster system is a giant, complex system, its corresponding disaster risk science naturally is a disciplinary group, which can be further divided into three pillars—disaster science, disaster technology, and disaster governance.

**Fig. 3:**
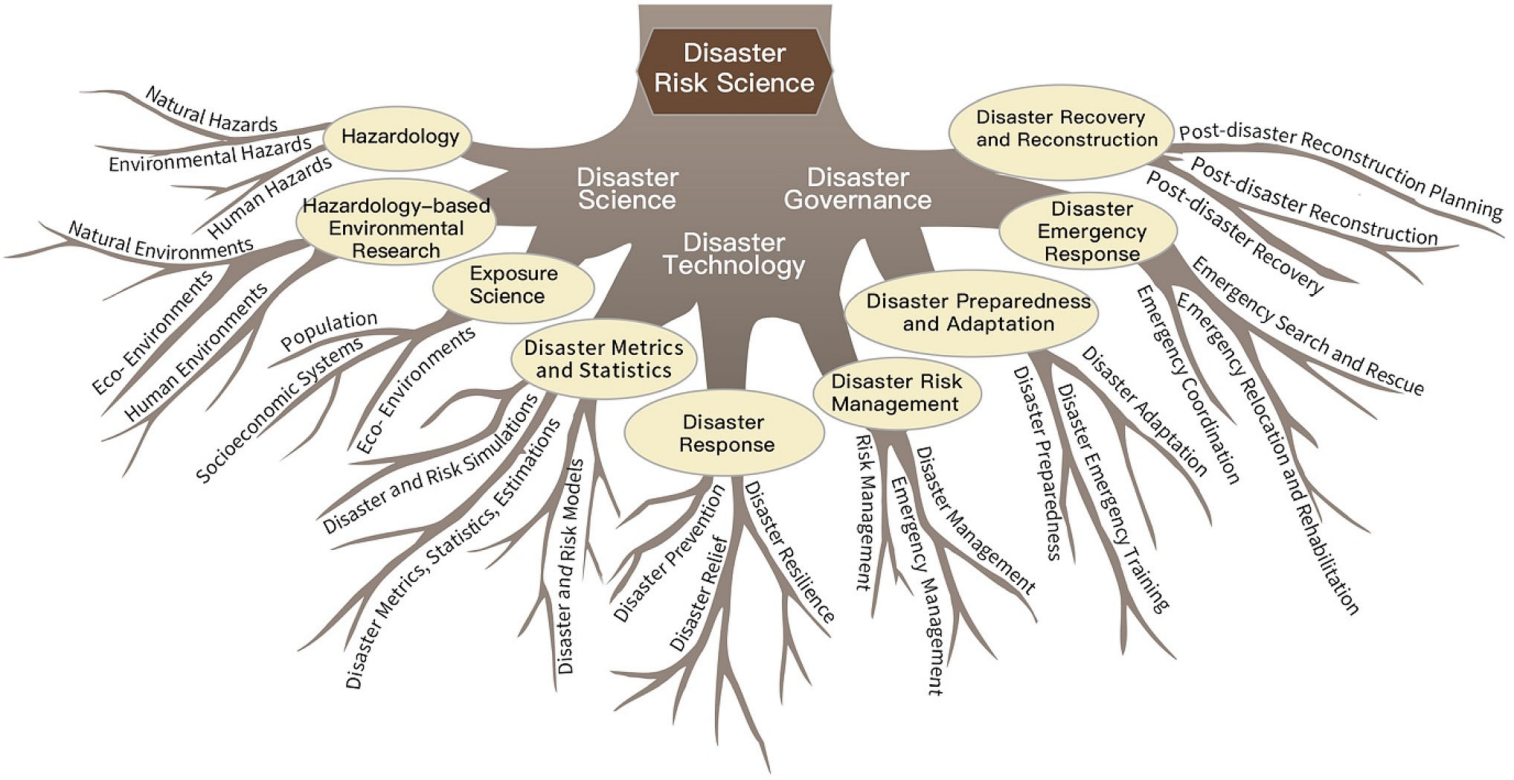
A framework of disaster risk science research—a root system diagram of the three-layered disciplinary structure

*Disaster science* mainly focuses on the structure, function, properties, and dynamics of disaster systems. Research on disaster systems’ structure includes the study of hazards, socioeconomic exposure, and the environment. Research on the function of disaster systems includes studies on hazardousness (threat or danger), socioeconomic vulnerability, and environmental stability. Research on the properties of disaster systems refers to studies that explore their interconnectedness, regionality, complexity, and coupling features. Research on disaster system dynamics refers to the mechanism, processes, and evolution of these systems. Given these topics, disaster science can be further divided into fields such as hazardology, hazardous environment studies, and exposure science (Fig. 3).

*Disaster technology* mainly focuses on the R&D of disaster systems’ metrics and statistics, disaster response technologies, disaster and risk management technologies, and technology integration. Disaster system metrics and statistics includes disaster monitoring (site and station based, or using remote sensing data and big data), forecasting, early warning, loss estimation (statistics and relative impact), disaster modeling (experimental, numerical, and scenario modeling of mechanisms and impacts), disaster risk assessment (quantitative, semiquantitative, qualitative), and disaster risk simulation. Disaster response technology includes technologies for prevention (inventory, maps, regionalization, plans and planning, fortification standards, insurance), resilience (structural measures, retrofitting, relocation, site improvement), and relief (rapid assessment, aid to victims, organizing assistance and funding, distribution of material and funds), and their integration. Disaster and risk management technology covers disaster management (regulation, standard, procedure, method, statistical indicator system, mapping, logistic, online service and automation, information system), disaster risk management (regulation, standard, procedure, method, mapping, premium setting, loss assessment, insurance, financing, online service and automation, information system), and disaster emergency management (regulation, standard, procedure, method, plan, logistic, institution, online service and automation, information system) and technologies. Disaster technology can be divided into disaster metrics and statistics, disaster response technology, and disaster and risk management technology (Fig. 3).

*Disaster governance* mainly focuses on developing approaches and methods of preparedness, adaptation, emergency response, recovery and reconstruction, and their integration. Disaster preparedness and adaptation management includes capacity building for the management of inventories, shelters, and command system; disaster response schemes, plans and standards, and disaster response media communication, campaign, and education. Emergency response management covers emergency command and rescue systems, logistics and allocation of goods and materials, and joint defense and control. Recovery and reconstruction management covers rehabilitation, carrying capacity assessment, ground safety assessment, and reconstruction planning. According to the topics/fields mentioned above, disaster governance can be divided into disaster preparedness governance, disaster emergency governance, and disaster recovery and reconstruction governance. Therefore, disaster risk science as an interdisciplinary group contains three pillars, nine core-areas, and 27 research fields (Fig. 3).

Disaster risk science has strong multi-, cross-, and transdisciplinary features. Its theory, methodology, technology, and governance systems must be broadly developed from the perspectives of science and engineering, as well as the humanities and social sciences. Therefore, disaster risk science, as a super disciplinary group, requires its researchers and students to have a wide knowledge background in the Geosciences (Geology, Geography, Atmospheric Sciences, Oceanology, Geomathematics, Geophysics, Geochemistry, Geometrics, Digital Earth, and so on), Life Sciences (Biology, Ecology, Medicine, Pharmaceutical Science, and so on), Economics, Management Science, as well as Mathematics, Physics, Chemistry, Information Science and Technology, Language and Literature, History, Philosophy, Sociology, Political Science, and Law, among others.

## Frontiers in the Disaster Risk Science Research

The Sendai Framework has outlined four priorities of actions, and these four priorities all require strong support from disaster risk science. This offers new opportunity but also challenges for disaster risk science. The UNISDR Science and Technology Conference on the Implementation of the Sendai Framework for Disaster Risk Reduction 2015–2030 was held in Geneva in 27–29 January 2016 (Dickinson et al. 2016). Scientists, policymakers, business people, and practitioners at the conference focused on following key questions: (1) In what way would the UNISDR Science and Technology Partnership leverage local, national, regional, and international networks and platforms to advance multidisciplinary research and bring together science, policy, and practice? (2) How is disaster risk understood, and how are risks assessed and early warning systems designed? (3) What data, standards, and innovative practices would be needed to measure and report on risk reduction? (4) What research and capacity gaps exist and how can difficulties in creating and using science for effective disaster risk reduction be overcome? In support, UNISDR also revised DRR-related terminology, and set up monitoring indices for the seven targets listed in the Sendai Framework. In light of these updates, five topics should be addressed as high priority research areas: Dynamics and non-dynamics of disaster systems; disaster response digital systems; disaster response models; integrated disaster risk governance paradigms; and new disaster emergency and risk management systems.

### Dynamics and Non-dynamics of Disaster Systems

Disaster systems are typical coupled human–environment systems, or socioecological systems, with the features of giant systems and complex network systems. A regional disaster system can have complex network system behaviors such as disaster swarms, disaster chains, and disaster compounds (Shi, Lu, et al. 2014). A disaster swarm refers to the phenomenon that disasters often occur as spatial and temporal clusters (Shi 1991). It is close to the concept of multi-hazards, and this clustering property mainly depends on the environment of the region. A disaster swarm could be further grouped into a temporal co-occurrence and a spatial cluster of hazards. Disaster chains (or cascading disasters) refer to the triggering or causal relationship between one disaster and other disaster(s). It can be further divided into parallel disaster chains (one-to-many; or ripple behavior), and sporadic disaster chains (one-after-another; or the domino effect) (Shi 1991; Shi, Lu, et al. 2014). The concept of a hazard/disaster compound was proposed by Hewitt and Burton, and indicates “the co-occurrence of multiple disasters that could induce social risks” (Burton et al. 1993; Hewitt 1997). In the Intergovernmental Panel on Climate Change (IPCC) framework, hazard compounds are a special case that result when two or more climate extremes occur together. We have framed disaster compound as the case in which two or more disasters without any causal relationships have occurred simultaneously or consecutively, and induces much larger consequences than the simple summation of each disaster, even if they are not extremes when considered separately (Shi, Lu, et al. 2014). Understanding these complex features of disaster systems is important for further understanding the formation process of hazards and disasters.

The existing literature has obtained some understanding of disasters induced by climate extremes. Knowledge gaps still exist, however, about the complexity of global change, particularly about the impact of climate change on disaster systems. Our earlier studies have shown that climate change impact on disaster has three different components: (1) the trade-off induced by climate trends; (2) uncertainty introduced by climate variability; and (3) the extreme impact associated with climate extremes (Shi, Ye, et al. 2012; Wang et al. 2018). The trade-offs induced by climate trends depend largely on geographical location. For instance, for crops grown in higher latitudes or altitudes, warming climate brings more potential gains than losses. By contrast, in middle-and-lower latitude arid and semi-arid regions, warming would further exacerbate drought, making it even more difficulty to reduce agricultural risks. The impact of climate variability largely depends on the threshold of triggers. Variation of precipitation and temperature without exceeding the prevention capacity of human society could have some effect, but not disaster. Once variations in climate exceed impact prevention capacity, a tipping-point might occur and catastrophic extreme climate and weather disaster could be triggered, causing huge losses and long-run impacts.

Studying multi-hazard, disaster chain, and disaster compound occurrences, and the impacts of climate change in its trend, variability, and extremes have important theoretical and practical meanings in understanding regional hazard mechanisms and disaster processes. Presently, we have only started to study the many features of disaster system dynamics and non-dynamics, that is, their interconnectedness, regionality, complexity, and coupling. In most cases, the existing literature considers one or two features at one time, and mainly focuses on single hazard types. Studies on the dynamics of multi-hazard, disaster chain, and disaster compound events have been very limited. Current studies on the impact of climate change on disasters paid more attention to the impact of climate trend, that is, estimation of loss and of the impacts of global average temperature increase, than that of the changes in climate variability and extremes. Studies about the dynamics of climate change in its mean, variability, and extremes, together forming systemic risk, are also limited. Study of the dynamics of disaster systems continues to rely heavily on complex network system dynamics. There is an urgent need to further establish novel quantitative indices, and deepen our understanding of the mechanisms and processes that are basic to network system dynamics. Study on the non-dynamics of disaster systems, that is, disaster risk related management and policy issues, have largely been limited to statistical analysis.

The globalization process has further highlighted the regional, interconnective, coupled, and complex features of disaster systems. The system dynamics and non-dynamics of disaster systems not only reveal the “node degree” behavior of their elements, but also their “consilience” behavior (Hu et al. 2017). The consilience of disaster systems can reflect the differences of regional disaster systems not only in their mechanisms and processes, but also the integrated features of their structures and functions. The concept of consilience also makes quantitative analysis and simulation of disaster systems possible. Disaster system dynamics contain mechanisms, processes, and dynamics models. Numerical simulations together with statistical models have often been applied to model the nature of nonlinear dynamic processes. The non-dynamics processes—disaster and disaster risk management schemes and policies—of a disaster system, like many other human–environment systems, have limited quantitative indicators and data to model, and mostly relied on statistical models. The integration and coupled study of the system dynamics and non-dynamics features of disaster systems have always been a tough challenge in disaster risk science research. With the development of the supercomputer, big data, artificial intelligence, visualization, and modern 5G network systems and their application in coupled human–environment systems, a promotion in the integration and coupled study on the system dynamics and non-dynamics of disaster systems is expected, making possible a deeper understanding of the formation processes of hazard and disaster.

### Disaster Response Digital Systems

Information systems have played important roles in disaster response. The digital system for disaster response is a critical part in digital Earth systems, including the disaster response system, digital disaster system, and modeled disaster system.

The disaster response system is the system that describes the response of a regional DS at varying scales—community, local place, nation, subregion, region, and the globe—to individual disaster events and regional disasters. These include various types of response activities, such as DRR demonstration communities,^1^ the UNDRR (United Nations Office for Disaster Risk Reduction) disaster resilience scorecard for cities,^2^ WHO-international safe community approach to injury prevention,^3^ and various other types of resources for disaster response.

The digital disaster system is the data center of a regional disaster system, mainly about the construction of and quality standard for information products. Disaster system data centers can be established by expanding the databases created from implementing the *Yokohama Strategy*, the *Hyogo Framework for Action*, and the *Sendai Framework for Disaster Risk Reduction,* and incorporating new data obtained from new technologies and approaches such as earth observation, internet resources, big data processing, and supercomputing. The goal of developing such digital disaster systems and data centers is to turn the observation of individual disaster events from occasional observation into long-term and fixed-site observation, from static observation to dynamic analysis, from human observation to artificial intelligence-supported observation. In this way, it is possible to provide critical data support and management services for global and regional DRR. Such data centers must be capable of receiving data from local sources and the cloud, accessing telecommunication, navigation, and remote sensing data, and assimilating multisource, spatial–temporal data.

The modeled disaster system is the modeling platform for quantitative studies of regional DS mechanisms, processes, and dynamics, loss estimation and modeling for disaster events, disaster risk assessment, and simulation of regional disasters. Based on the support of the disaster response system and data centers, technologies such as cloud computing, geographic information systems, big data visualization, virtual reality and augmented reality, and artificial intelligence can be applied to conduct all-weather, full-element, whole-process, and all-scale integrated simulation via various types of disaster and disaster-risk models.

### Disaster Response Models

Disaster response models include those designed models for individual disaster events and for regional disaster systems.

*Systematic response model for individual disaster events* From the disaster management cycle (Carter 2008), the management of individual disaster events includes the stages of preparedness, prediction and early warning, emergency response, relief, recovery and reconstruction. The Sendai Framework divides the response to a disaster event into five stages: preparedness, emergency, rehabilitation, recovery, and reconstruction (UNISDR 2015). We divide the response to an individual disaster event into three phases: pre-disaster, during-disaster, and post-disaster. These three phases cover the preparedness, emergency response, recovery, and reconstruction stages that form the functional system of integrated disaster risk governance. Of the four stages, preparedness is the key. The response of China to the 2020 novel coronavirus has revealed the drawbacks of an insufficient resources reserve and the weakness in existing prediction and early-warning capability.

*Systematic response model for regional disasters* At the regional scale, disaster response has to strive for a synthesis of prevention, resilience, and relief, with a major focus on prevention. This is also the structural system of integrated disaster risk governance. Prevention is the key in regional disaster response. From the practice of disaster response, prevention refers to the set of measures that include peril identification and survey, disaster governance regionalization, prevention standard determination, and disaster insurance development. The key for resilience includes infrastructure construction, and retrofitting. The key for relief includes rapid disaster assessment and humanitarian aid. The response system for regional disasters is closely related to the developmental disaster risk governance paradigm (see below for more discussion). The efficiency and cost-effectiveness of a response system for regional disasters can be improved via optimization under the rules of effectiveness, efficiency, and equity (Shi 2011b; Hu et al. 2014).

### Integrated Disaster Risk Governance Paradigms

Presently, there are plenty of ongoing discussions on the synergetic paradigm of green development and DRR, the collaborative paradigm of regions and sectors to increase DRR resources utilization efficiency, and the consilience paradigm of stakeholder involvement to improve DRR resources utilization effectiveness. The ultimate goal of these paradigms is to improve the efficiency and effectiveness of integrated DRR resources utilization (Hu et al. 2014; Shi 2018).

**Fig. 4:**
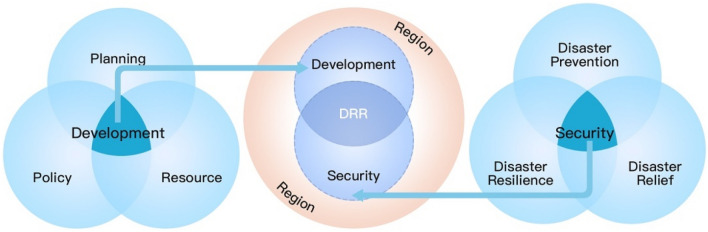
Synergetic paradigm for disaster risk governance and sustainable development. *Source* Adapted from Shi (2008b)

*Synergetic paradigm* The synergetic paradigm achieves the balance of development and security (Fig. 4) via effective disaster risk reduction and an overall plan of green development and integrated disaster risk governance in order to promote sustainable development (Shi 2008b). This process is also referred to as managing risk for development (World Bank 2014). It is characterized by the following goals: (1) coordinate the establishment of a resource-saving and environment-friendly society, the promotion of green economy, and progress toward a circular economy; (2) enhance the administrative functions of governments at all levels, and promote the roles of other stakeholdes (entrepreneurs and households) in integrated disaster risk governance (Shi et al. 2006); (3) increase integrated disaster risk governance resource utilization efficiency and effectiveness, optimize the coordination of DRR plans at different levels and sectors, synergize innovative development that is coordinated, green, open, and shared, and that promotes the establishment of “win–win” models for all. The establishment of regional integrated disaster risk governance synergetic paradigm has important supportive roles in improving response capability to regional disasters.

**Fig. 5:**
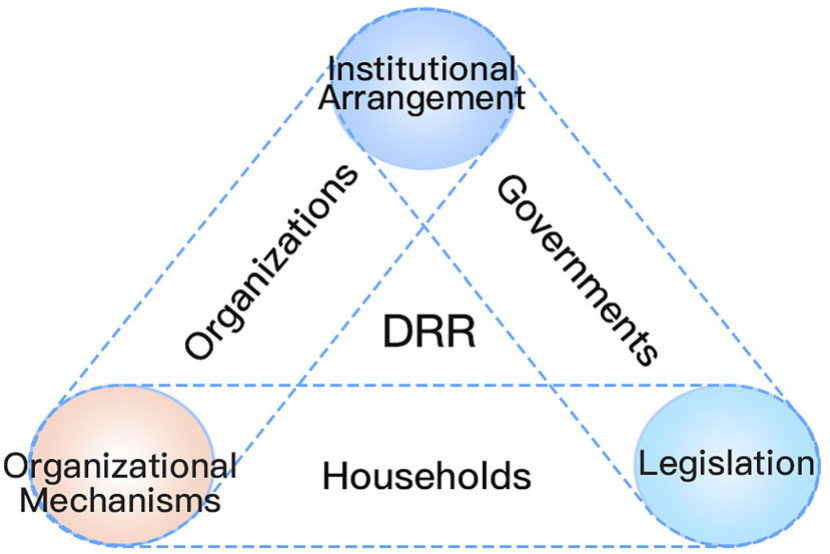
Collaborative paradigm for regional disaster risk governance: stakeholders’ relationship

*Collaborative paradigm* The collaborative paradigm is important in improving the stakeholders’ role in regional integrated disaster risk governance, particularly their capability to respond systematically in individual disaster events. The collaborative paradigm attempts to build up the cooperative relationship of stakeholders in the system via improvement of institutional arrangements, operational mechanisms, and legislation (Shi 1996, 2009) (Fig. 5). The establishment of a regional disaster risk governance collaborative paradigm can effectively guide the improvement of response capability in event-based disaster management systems (Shi et al. 2006).

*Consilience paradigm* The consilience paradigm involves the integration of “cohesion” and “joining force,” which is the integration of hard and soft power, coupling of the dynamic and non-dynamic elements of systems, and integration of structural and nonstructural measures in the response of stakeholders (governments, the private sector, and individuals and households) to individual disaster events and regional disasters in integrated disaster systems. Consilience is a metrics and description of the “cohesion” and “joining force” capabilities, and is related to system structure and function. In the concept of consilience, cohesion refers to the process by which system components reach consensus, and joining force refers to the process that links system components to form joint forces. Consensus is reached and joint forces are formed in order to resist gradual or sudden hits from external hazards. Consilience refers to four different synergetic principles in disaster management: (1) tolerance (cohesion), namely “united people can move mountains” (traditional Chinese saying, *Ren Xin Qi, Tai Shan Yi*), “united people are strong” (*Qi Min Zhe Qiang*); (2) constraint (cohesion), namely “sacrifice a pawn to save a castle” (*Qi Zu Bao Ju*), “triumph comes when leaders and followers share the same goal” (*Shang Xia Tong Yu Zhe Sheng*); (3) amplification (joining force), that is, “more hands produce a stronger flame” (*Zhong Ren Shi Chai Huo Yan Gao*); and (4) diversification (joining force), that is, “ten chopsticks are stronger than one.” Consilience is closely related to the vulnerability, resilience, and adaptability of disaster systems (Shi, Wang, et al. 2014). Consilience can be computed by using consilience degree (Hu et al. 2017). Simulation experiments have shown that a higher consilience degree indicates a greater capability to resist external shocks. Optimization over consilience degree can substantially increase DRR resources utilization efficiency and effectiveness (Hu et al. 2017).

### New Disaster Emergency and Risk Management Systems

In the era of globalization, modern disaster risks have wide spatial extent, stronger systemic features, and greater uncertainty and unpredictability than ever before. Disaster risk is no longer a matter of single, one-shot events, but a new societal norm. We have entered the “risk society,” and face the situation of “living with risk” (Beck 1999; UNISDR 2004). It has become an urgent issue in DRR to establish new emergency and risk management systems, which is also a new challenge in disaster risk science research.

*New disaster emergency management systems* An emergency management system is an important part of the disaster response system and the core of individual disaster event response. Traditional emergency management systems, which consist of ex-ante prevention, during event coping, and ex-post recovery, have been challenged by multi-hazards, disaster chains, and disaster compounds. It has been an arduous task for disaster risk science research to reveal how we can improve the emergency management capacity against large-scale disasters by regrouping disaster management administrations, establishing new schemes, improving legal systems, encouraging applied S&T as well as R&D, restructuring educational and cultural systems, enhancing rescuing systems, empowering social mobilization, and improving guaranteed emergency resources access. New disaster management schemes, administrations, systems, and command and rescue forces must be established to save people’s lives and property from disasters by using science, technology, planning, and management measures. During the response to the 2020 novel coronavirus outbreak, the Chinese government has called for a new emergency management strategy of “strengthening confidence, working together, scientific prevention and control, and targeted implementation” (Zhao et al. 2020).

Globally emergency management systems differ substantially by nations’ administrative system, institutional arrangement, and legal system. China has adopted the emergency management system of “centralized leadership, integrated coordination, management by category, multi-level responsibility, and jurisdiction management,” which places more emphasis on the regional integration of different government authorities. The United States adopted the dual-core (Federal and State administration) system supported with Federal disaster-related agencies, which emphasizes strongly the role of responsible agencies. Japan adopted a system of centralized management with the participation of local governments and departments. Since 1994, Russia has set up its Ministry of Emergency Situations. The department takes full responsibility for commanding and coordinating emergency situations, and reports to the President directly. A successful emergency management system requires an authoritative command system, solid legal support, strong rescue teams, extensive social mobilization, efficient joint defense and joint control mechanisms, strong S&T support, a consilient social environment, and timely and accurate online information services. It requires practical tests to identify which system could be more efficient, and which system could be more effective for various types of emergencies.

*New disaster risk management system* The UNDRR has emphasized the development strategy of “living with risk.” The dependence and interactions between different types of disaster risks are intensifying in the era of globalization. The occurrence of disaster chains becomes more frequent, with more complicated mechanisms and larger scale impacts than before. Traditional disaster risk management systems based on quantitative measurement and assessment, and an expertise system of single disciplines, are facing a series of challenges stemming from multi-hazards, disaster chains, disaster compounds, and global change. A new disaster risk management system must follow the overall trend in the “risk society.” There are different interpretations of disaster management and disaster governance. In UNDRR’s perspective, disaster management refers to specific actions in DRR, while disaster governance emphasizes institutional arrangements. The International Risk Governance Council (IRGC) has advocated a change from risk management to risk governance. The IRGC proposes to integrate DRR with green development. The Chinese government is promoting holistic national security, that is, an effort to strive for people-centered and coordinated development, prevention-centered and integrated DRR, response with legal and S&T support, and private-sector participation under government leadership. In an era of globalization, more comparative studies are needed to understand how to establish a brand-new disaster risk management system (Hu et al. 2017).

Integrated disaster risk governance needs to pay special attention to modern coupled human–environment system research (Liu et al. 2007) and the human dimensions of disaster vulnerability research (Cutter and Finch 2008). This field could borrow the idea of the Dujiangyan irrigation system from ancient China so that appropriate modification to the local environments can lead to the win–win result of risk reduction and development gains (Yan et al. 2017). It could also consider China’s response strategy to large-scale disasters, that is, to focus on stakeholder cohesion and joining forces, conduct joint defense, and centralize community prevention and governance in order to strengthen confidence, work together, scientifically prevent and reduce disaster risks, and achieve targeted implementation of policies.

## Conclusion

It has been 30 years since UN lunched the IDNDR and the global joint efforts devoted to reducing disaster risk. There has been remarkable development in DRR science and technology. In the present article, we have reviewed the framework and contents of disaster risk science research and summarized recent progress in disaster risk science with regard to hazard study focusing on the physical mechanisms, disaster study concentrating on estimating and modeling of losses, risk study emphasizing assessment, and disaster response study highlighting actions for risk reduction. Based on these understandings, we propose a three-dimensional and three-layered disciplinary system for disaster risk science research containing three pillars (disaster science, disaster technology, and disaster governance). Key research frontiers in this field, including the dynamics and non-dynamics of disaster systems, disaster response digital systems, disaster response models, and integrated disaster risk governance paradigms are also briefly discussed.

## Footnotes

1 https://openknowledge.worldbank.org/handle/10986/34090, Chapter 4.

2 https://www.undrr.org/publication/disaster-resilience-scorecard-cities.

3 https://pubmed.ncbi.nlm.nih.gov/19588359/.
